# Associations of Self-Efficacy, Optimism, and Empathy with Psychological Health in Healthcare Volunteers

**DOI:** 10.3390/ijerph17166001

**Published:** 2020-08-18

**Authors:** Alberto Dionigi, Giulia Casu, Paola Gremigni

**Affiliations:** 1 Italian Federation of Clown Doctors (FNC), via Rovescio 2185, 47023 Bagnile di Cesena (FC), Italy; albe.dionigi@gmail.com; 2 Department of Psychology, University of Bologna, viale Berti Pichat 5, 40127 Bologna, Italy; giulia.casu3@unibo.it

**Keywords:** volunteers, self-efficacy, optimism, empathy, psychological well-being, subjective well-being, hierarchical regression analysis

## Abstract

Optimism and self-efficacy have been associated with psychological health. Empathy has also been found to have a unique role in community health volunteering and promote positive functioning. This study investigated whether self-efficacy and optimism were associated with psychological health in terms of psychological and subjective well-being in healthcare volunteers. It also investigated whether empathy added to the explanation of psychological health, over and above that accounted for by self-efficacy and optimism. A convenience sample of 160 Italian clown doctors volunteering in various hospitals completed self-report measures of self-efficacy, optimism, empathy, psychological well-being, and subjective well-being. Results of hierarchical multiple regression analysis indicated that self-efficacy and optimism were associated with both outcomes and that aspects of empathy, such as others’ perspective taking and personal distress for others’ difficulties, added to the explanation of psychological health with opposite effects. The present study adds to previous research on the role of self-efficacy, optimism, and empathy for community health volunteers’ psychological health. It also offers suggestions regarding the training for this type of volunteer.

## 1. Introduction

Volunteering is an intentional, proactive helping behavior aimed at enhancing social capital, strengthening the community, and delivering services that otherwise would have costs or be underprovided [1,2].

In the last three decades, many volunteers have approached the clown activity in healthcare settings since it was found to humanize healthcare and promote patients’ good health [3,4]. Clowns are colloquially called “clown doctors” as they are identified as part of the hospital medical staff, although they are not necessarily medically qualified [5].

The central aspect of volunteering is the free will to help others [1,2]. However, research has shown that people are motivated to volunteer also to grow and satisfy personal psychological needs [6,7,8]. Evidence exists that volunteering may promote the volunteers’ benefits in terms of physical, social, and psychological health [9,10,11,12,13,14,15]. Most studies have focused only on the beneficial effects of engaging in volunteering, whereas individual characteristics that may contribute to volunteers’ psychological health have not been thoroughly investigated. We committed to filling this gap in the literature by examining whether some factors associated with positive mental functioning of other populations may also play the same role in healthcare volunteers like clown doctors.

Multiple factors have shown to contribute to individual psychological health; among them, we were interested in self-efficacy, optimism, and empathy.

Psychological health has been referred to as a variety of different constructs. Positive psychology researchers differentiated between eudaimonia and hedonia [16], which are referred to and assessed as psychological well-being and subjective well-being. Psychological well-being is the realization of one’s authentic self and positive functioning in several domains: self-acceptance, positive social relations, autonomy in thought and action, meaning and purpose in life, and continuous growth as a person [17]. Subjective well-being is a person’s positive cognitive and affective evaluations of her or his life, including satisfaction with life and the experience of positive emotions and mood states [18,19]. Since psychological and subjective well-being refer to different constructs, it was worth considering both as outcomes.

Self-efficacy and optimism are relatively stable cognitive traits that are distinct yet similar in some respects [20]. They are both related to positive expectations in life and help people better address the difficulties [21,22,23,24]. Self-efficacy refers to the individuals’ beliefs about their capability to produce given attainments and perform specific tasks [25]. People high in self-efficacy tend to deal effectively with a variety of stressful situations to achieve their goals [26,27,28]. Optimism refers to the generalized expectation of positive vs. adverse outcomes in essential domains of life [29,30]. As a consequence of their positive view of the future, people with optimistic traits, compared to those with pessimistic traits, are more likely to use active coping strategies, achieve high goals, and engage in social activities [31,32,33]. Extensive research has documented the link of self-efficacy and optimism with positive psychological health. Self-efficacy was positively associated with subjective well-being [34] and its components like positive affect [35] and life satisfaction [36]. It was also positively related to subjective well-being in nurses [37] and psychological well-being in nursing home residents [38]. Optimism was a significant predictor of volunteerism, which positively influenced life satisfaction and psychological well-being [39]. A recent study found a direct positive effect of optimism on subjective well-being [40]. A meta-analysis found that optimism had moderated to strong associations with the three aspects of subjective well-being: it was associated positively with positive affect and satisfaction with life and negatively with negative affect [41].

Empathy is an individual’s tendency to empathize with others, which develops first in childhood and remains relatively stable throughout the lifespan [42,43,44]. However, whereas personality traits are relatively fixed, there is evidence that empathy may be influenced by education and training [45]. Davis [42] identified an affective and a cognitive component of empathy. The affective component includes empathic concern, which involves feelings of sorrow for others’ misfortune, and personal distress, which involves feelings of suffering for others’ difficulties. The cognitive component refers to perspective taking as the ability to accurately imagine and adopt others’ point of view. Globally, both empathic concern and perspective taking components have been linked to psychological health [46,47]. A study of older adults’ informal caregivers showed that those with greater cognitive empathy appraised the caregiving situation as less stressful and threatening; in contrast, caregivers with lower empathy had reduced well-being and more depressive symptoms [48]. In the healthcare setting, greater empathic concern and perspective-taking were associated with higher healthcare professionals’ psychosocial well-being. In contrast, higher personal distress in the presence of another’s suffering was linked to greater emotional exhaustion and risk for burnout [49,50,51].

According to the empathy-altruism model [52], empathy may lead to engaging in prosocial behavior by evoking empathic feelings, which elicit altruistic motivation and interest in others’ well-being. Therefore, it is a crucial component in the decision to volunteer [6,46], and greater empathy was positively associated with the amount of time spent on volunteering and the duration of the voluntary service [53]. Although the direct link between volunteers’ empathy and their psychological health has not been investigated yet, we could expect that empathy cognitive and affective components were associated with volunteer clown doctors’ psychological health in the same direction as healthcare professionals [49,50,51]. Given the crucial role of empathy in volunteering, we also expected that empathy adds to the explanation of psychological health, over and above that accounted for by self-efficacy and optimism.

This study aimed to test whether self-efficacy and optimism were positively associated with volunteer clown doctors’ psychological health and whether empathy explained some incremental variance of this outcome. Specifically, we expected that empathic concern and perspective taking were positively associated, while empathic personal distress was negatively associated with the volunteers’ psychological health. Our general intent was to add to how healthcare volunteers can benefit from their volunteering activities, also considering their characteristics.

## 2. Materials and Methods

### 2.1. Participants

Participants who completed the online survey were 160 volunteer healthcare clown doctors. This number represents about 3% of the Italian volunteer clown doctors active in healthcare settings. The participants’ age was between 20 and 60 years. The majority were female, almost half had a high school diploma, their length of experience as volunteer clown doctors varied from 1 month to 11 years, and about one third had no other volunteering experience before starting their clown activity. Participants’ characteristics are presented in Table 1.

### 2.2. Measures

Participants completed an online sociodemographic form including gender, age, education (secondary school or university degree), length of volunteer service (months), and previous volunteering experience (yes/no).

We assessed self-efficacy with the 18-item self-report Perceived Personal Efficacy for Members of Voluntary Associations (PPEV) [54]. This Italian scale measures the extent to which a member of a voluntary association feels capable of facing the challenges and critical events occurring during her or his volunteer activity (e.g., “I can handle the stress of my work as a volunteer”, “I can cooperate with my colleagues”). Responses are provided on a 5-point scale from 1 (entire disagreement) to 5 (entire agreement). An overall higher score corresponds to greater self-efficacy levels.

We measured optimism with the Italian version [55] of the Life Orientation Test—Revised (LOT-R) [56]. The LOT-R contains 10 items (e.g., “In uncertain times, I usually expect the best”, “If something can go wrong for me, it will”) rated on a 5-point scale from 0 (strongly disagree) to 4 (strongly agree). Four items are unscored fillers. Higher LOT-R total scores indicate higher optimism.

We measured empathy with the Interpersonal Reactivity Index (IRI) [42] in its Italian version [57]. The IRI is a 28-item multidimensional measure of four 7-item components: Fantasy (IRI-Fs) as the tendency to identify with fictitious characters (e.g., “I really get involved with the feelings of the characters in a novel”); Perspective Taking (IRI-PT) as the ability to adopt the perspective of others (e.g., “I try to look at everybody’s side of a disagreement before I make a decision”); Empathic Concern (IRI-EC) as the tendency to experience feelings of compassion and sympathy for others in need (e.g., “I am often quite touched by things that I see happen”), and Personal Distress (IRI-PD) as the proneness to feel uncomfortable about the distress of others (e.g., “When I see someone who badly needs help in an emergency, I go to pieces”). Items are rated on a 5-point scale from 0 (does not describe me well) to 4 (describes me very well). Higher subscale scores indicate greater empathic tendencies.

We measured psychological well-being with the 18-item Psychological Well-Being Scales (PWBS) [17], which refer to positive psychological functioning in terms of self-acceptance, environmental mastery, quality relationships, growth and development, purposeful living, and autonomy. Items (e.g., “For me, life has been a continuous process of learning, changing, and growth”) are rated on a 6-point scale from 1 (completely disagree) to 6 (completely agree). We used the Italian version [58], with a higher global score indicating greater psychological well-being.

We measured subjective well-being with the Five-Item World Health Organization Well-Being Index (WHO-5) [19], a 5-item self-report measure of positive feelings in the last two weeks (e.g., “I have felt cheerful and in good spirits”). Items were rated on a 6-point scale from 0 (none of the time) to 5 (all of the time), with a higher global score indicating greater perceived subjective well-being.

### 2.3. Procedure

The present study was cross-sectional and involved healthcare volunteer clown doctors. The criteria for being included in the study were having at least one month of experience as a volunteer healthcare clown and being older than 18 years. The only exclusion criterion was being a professional clown doctor since this study targeted healthcare volunteers. The National Federation of Clown Doctors (FNC) Ethics Board approved the study (FNC-01/2017), which respected the Declaration of Helsinki ethical principles. The FNC brings together a large number of Italian associations of clown doctors. In Italy, clown doctors active in healthcare settings are approximately 6000, with a prevalence of volunteers. However, a precise census of this population at the national level is not available.

We created an online battery for this study; the first page of the survey contained an informed consent statement that described the research and ensured the participants’ anonymity. Only after clicking the “Yes, I consent to participate” button could respondents access the online questionnaire. We posted a link to the online battery on the FNC social network site (i.e., Facebook), which has more than 6000 followers, introduced by a brief description of the research and a clear indication of the inclusion and exclusion criteria.

### 2.4. Data Analysis

The reliability of measures was calculated using Cronbach’s alpha with an acceptable value ≥0.70. Descriptive statistics were calculated as frequency, mean value, standard deviation, and range according to categorical or continuous data. The objective of this study was tested using multiple linear regression analysis. We preliminarily examined the predicted probability (P-P) plot to determine if the residuals were normally distributed. We check the assumption of homoscedasticity by plotting the predicted values and residuals on a scatterplot. Levels of multicollinearity were examined by the variance inflation factor (VIF). A general rule of thumb is that VIFs >4 warrant further investigation, while VIFs >10 indicate serious multicollinearity. Pearson’s bivariate correlations or analyses of variance (ANOVAs) were performed to select the independent variables for the regression models based on their significant associations with the dependent variables. Hierarchical multiple linear regression analysis was used to examine the effects of self-efficacy and optimism on psychological and subjective well-being, and the incremental impact of empathy on the outcomes, over and above that contributed by self-efficacy and optimism. Two separate models were tested with PWBS and WHO-5 total scores as outcome variables. For each model, self-efficacy (PPEV) and optimism (LOT-R) scores were entered at the first step, and each of the empathy (IRI) subscales was introduced at the subsequent steps. The significance level was set at *p* ≤ 0.05. Interpretation of effect sizes was based on Cohen’s [59] recommendations, with Pearson’s *r* or standardized beta (*β*) of 0.10 considered small, 0.30 medium, and 0.50 large, and *R*^2^ and *η*^2^ of 0.01 considered small, 0.09 medium, and 0.25 large.

Analyses were performed with the statistical package for social sciences IBM SPSS for Windows, Version 26.0, released in 2019 by IBM Corp., Armonk, NY, USA.

## 3. Results

### 3.1. Preliminary Results

The reliability of measures was acceptable with all the Cronbach’s alpha values ≥0.70 (Table 2). Table 2 presents the descriptive statistics of all the study psychological variables.

A check of assumptions for both regression models showed that the P-P plot’s residuals followed a normal distribution, and homoscedasticity emerged (i.e., residuals were equally distributed). VIFs varied between 1.10 to 1.49, indicating an absence of multicollinearity, despite slightly to moderated intercorrelations between some of the independent variables (see Table A1 for Pearson’s correlation coefficients).

**Figure 1:**
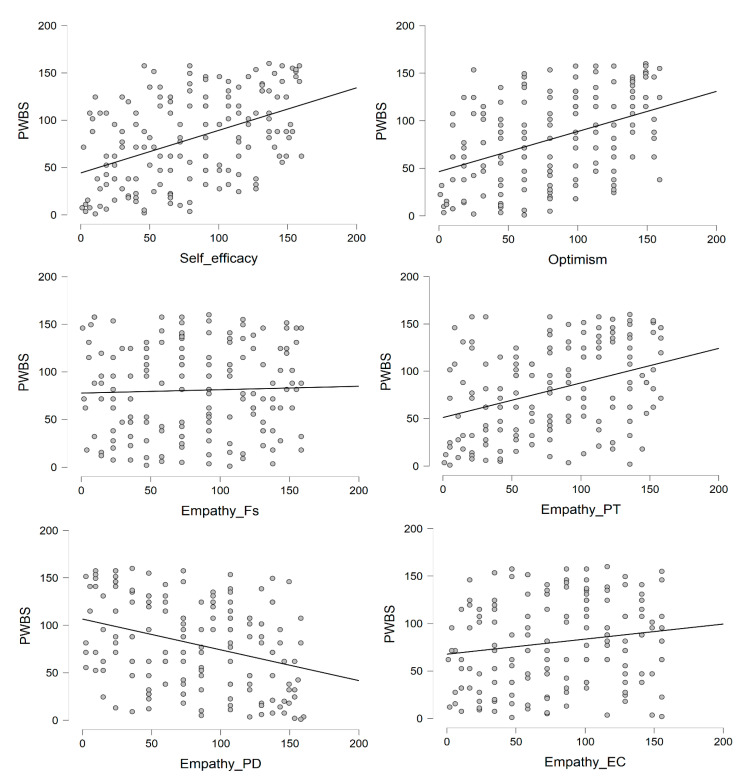
Scatterplots of correlations of psychological well-being with other psychological variables. PWBS = Psychological well-being; Fs = Fantasy; PT = Perspective Taking; PD = Personal Distress; EC = Empathic Concern.

**Figure 2:**
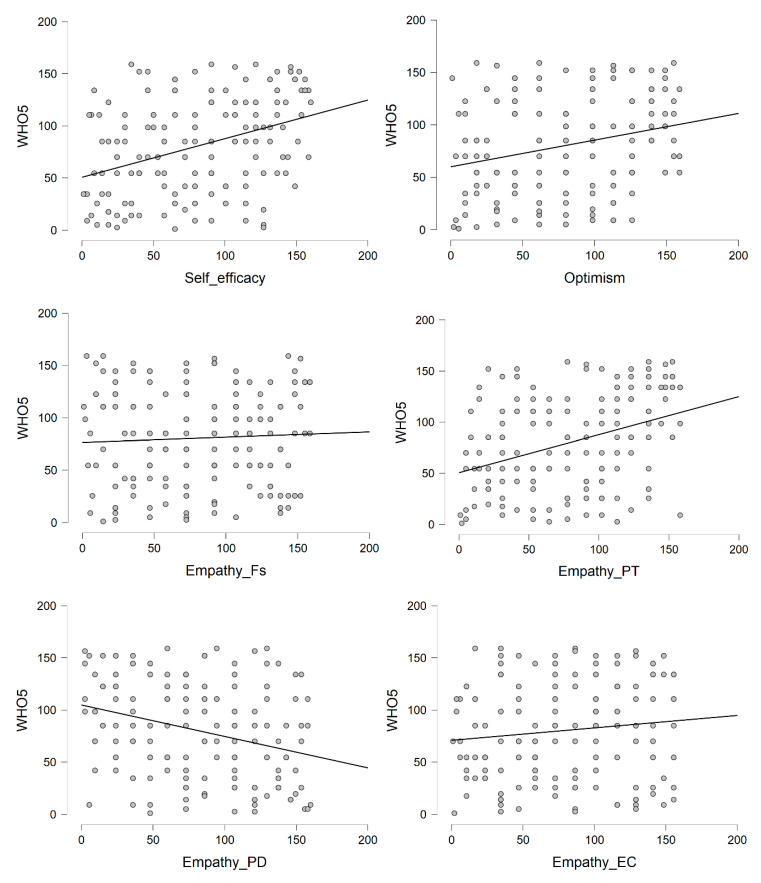
Scatterplots of correlations of subjective well-being with other psychological variables. WHO-5 = Subjective well-being; Fs = Fantasy; PT = Perspective Taking; PD = Personal Distress; EC = Empathic Concern.

Psychological (PWBS) and subjective (WHO-5) well-being were moderately intercorrelated (*r* = 0.41), showing that they were distinct dimensions of psychological health. Self-efficacy and optimism were significantly, moderately to-strongly correlated with PWBS and WHO-5 scores. Empathy was differently correlated with the outcomes depending on its dimensions: Fantasy and Empathic Concern were unrelated to both PWBS and WHO-5 and were thus excluded from the subsequent regression analyses. Perspective Taking and Personal Distress were significantly, moderately correlated with both outcomes, in a positive or negative direction, respectively (Figure 1 and Figure 2 and Table A1).

Sociodemographic variables (i.e., gender, age, education, length of volunteer service, and previous volunteering experience) were not significantly associated with PWBS and WHO-5; therefore, they were not controlled for in the regression analyses. Specifically, the correlations of age and length of experience with psychological and subjective well-being varied from −0.08 to 0.09 (*p* > 0.05). The corrected model (including gender, education, and previous volunteering experience) of ANOVA tests of between-subjects effects showed *F*(7) = 1.18 (*p* = 0.32) for PWBS, and *F*(7) = 0.86 (*p* = 0.54) for WHO-5, with nonsignificant contributions from any of the independent variables or interactions.

### 3.2. Regression Models

The two regression models included self-efficacy and optimism at step 1, empathic Perspective Taking at step 2, and emphatic Personal Distress at step 3 (Table 3). The results of the hierarchical multiple linear regressions are presented in Table 3. In the first regression model, with PWBS as the outcome variable, self-efficacy and optimism jointly explained a large portion (33%) of the outcome variability with medium effect sizes of their associations (standardized beta values) with the outcome. Adding Perspective Taking at the second step provided a significant, small improvement in the explained variance, which reached 35%. Adding Personal Distress at the third step, the explained variance further significantly increased to 36%. Standardized beta values of empathy subscales were significant, although small in effect sizes, with a positive sign for Perspective Taking and a negative sign for Personal Distress.

In the second regression model, with WHO-5 as the outcome variable, self-efficacy and optimism jointly explained a moderate portion (15%) of the variance. The magnitude of the association with subjective well-being was medium for self-efficacy and small for optimism. The introduction of Perspective Taking at the second step added a significant amount of explained variability, reaching 19% of total explained variance. The standardized beta value for Perspective Taking was significant, moderated, and seemed to suppress the effect of optimism that was not any more significant. Adding Personal Distress at the third step did not significantly improve the model, which explained 20% of the variability with a nonsignificant contribution of Personal Distress.

## 4. Discussion

This study aimed to test whether self-efficacy and optimism were associated with psychological and subjective well-being among volunteer clown doctors since they were found to be related to other populations’ psychological health [34,35,36,37,38,39,40]. This study also aimed to test whether empathy explained an incremental variability of psychological health, over and above self-efficacy and optimism since it is a crucial dimension in the decision to volunteer [6,46]. We chose clown doctor volunteers as they are required to be exceptionally able to empathically listen and respond to the emotional state of people, mostly children, in the hospital [5]. This was the first study investigating whether psychological characteristics of these volunteers can contribute to their positive psychological functioning.

This study’s findings indicated that self-efficacy and optimism explained part of the variability of the participants’ psychological and subjective well-being and that empathy explained an incremental variability. Self-efficacy and optimism jointly explained a moderate-to-large portion of the variability in positive psychological functioning, in line with previous evidence of such a positive role in other populations [27,28,41,60,61].

As for the contribution of empathy, the cognitive ability to imagine other people’s points of view explained an incremental 2% in the variance of psychological well-being and an additional 5% in the variance of subjective well-being. These findings were in line with evidence showing an association of the cognitive component of empathy with increased emotional adjustment in the general population and healthcare staff [46,50,51]. Perspective-taking involves a shift in the understanding of another’s situation as separate from one’s own; thus, it requires a self-other distinction that differentiates it from emotional contagion and is likely responsible for its positive association with psychological health. Notably, when empathic perspective taking was entered in the regression model with subjective well-being as the outcome, the contribution of optimism was suppressed, becoming nonsignificant. This result is coherent with experimental evidence that taking on others’ points of view can reduce individuals’ unrealistic optimism about the likelihood of specific life outcomes [62]. The affective component of empathy represented by personal distress also explained an incremental 2% of the variability in psychological well-being, over and above that explained by self-efficacy and optimism. In contrast, its association with subjective well-being did not reach statistical significance. In both models, more considerable empathic distress was linked to worse psychological and subjective well-being. It was consistent with previous research on healthcare professionals pointing to the potentially adverse implications of an individual’s tendency to experience negative affect involving vicarious arousal when exposed to another person’s plight [49,50,51].

Notably, in this study, only two of the four empathic dimensions considered contributed to explaining the variability of psychological health. Fantasy as the tendency to become immersed in the feelings and actions of fictional characters was unrelated to participants’ psychological health, consistent with previous studies [42,63]. Moreover, some researchers do not see fantasy as a core element of the empathic experience [44,46]. Instead, the nonsignificant association found in this study between empathic concern and psychological health is inconsistent with the majority of previous studies, which reported significant positive associations [46,50,51]. Such a result might be partly attributable to the peculiarity of our sample. Some researchers proposed an association between feelings of sympathy for the others’ misfortune and the willingness to get involved in volunteer work [42]. It is possible that for individuals with great empathic concern for others like volunteers involved in helping people, this component of empathy is not relevant to their mental health. However, further studies are needed to interpret these findings better.

As a final consideration, we should point out that although our regression models explained a large and medium proportion of the variability of psychological and subjective well-being, respectively, they did not fully explain the considered outcomes. Other studies of volunteers found other predictors of their psychological health, such as supportive work climate and autonomous motivation [64]. Other personality traits that characterize the Italian clown doctors, such as high agreeableness, conscientiousness, openness, extraversion, and low neuroticism [65], might also contribute to their psychological health. Therefore, further studies are needed to determine what could explain the remaining variance of psychological and subjective well-being.

### Limitations and Future Directions

The principal limitation of the present study was its cross-sectional design that did not allow conclusions about the causal effects of the participants’ characteristics on their psychological health. Further longitudinal studies are thus warranted to replicate our findings. It would also be desirable to replicate the study with professional clown doctors to investigate whether the associations found in this study can be extended. Another limitation is that we did not use measures of patients’ or hospital staff’s perspective on the clown doctors’ empathic skills. Future studies using multiple informants are thus encouraged. Also, among the independent variables, only self-efficacy referred explicitly to the volunteer activity; therefore, future studies might use measures of both optimistic and empathic tendencies related to volunteering. Finally, the findings of the present study cannot be generalized as the sample was self-selected. Thus, it was not representative of the Italian volunteer clown doctors, although the sample size included almost 3% of that population.

## 5. Conclusions

The present study adds to previous research on self-efficacy, optimism, and empathy for community health volunteers’ psychological health. It also highlights that empathy is a complex construct with multiple domains that can have different effects. While other-oriented perspective taking seems to have beneficial effects, self-oriented aversive emotional reaction to others’ suffering might have a negative impact.

Despite the cross-sectional nature of the present study, its findings might give some ideas for training volunteer clown doctors by focusing on specific dimensions of empathy. Although empathy has been conceptualized as a relatively stable tendency [42], there is evidence that it can be taught with effective training programs [45,66]. For example, engaging the volunteers in exercises to foster their ability to take the point of view of their assisted patients might promote potential benefits for their psychological health. On the other hand, improving their self-regulation abilities to reduce self-focused distress in response to needy people might prevent empathic over-arousal and preserve their mental health [6]. Further longitudinal studies are needed to evaluate the potential benefits of such training on the volunteers’ psychological health.

## Figures and Tables

**Table 1:** Participant characteristics (N = 160).

	N (%)	Mean (SD)	Range
Gender			
Female	102 (63.7)		
Male	58 (36.3)		
Age		32.5 (9.5)	20–60
Educational level			
Secondary school	81 (50.6)		
University degree	79 (49.4)		
Length of experience ^a^, months		42.4 (28.6)	1–132
Previous experience ^a^			
None	55 (34.4)		
Any	105 (65.6)		

SD = standard deviation; ^a^ = with reference to healthcare volunteer clowning.

**Table 2:** Descriptive statistics of psychological variables.

	Mean (SD)	Range	Cronbach’s
Self-efficacy (PPEV)	71.47 (8.82)	46–92	0.86
Optimism (LOT-R)	16.88 (3.67)	2–24	0.71
Empathy—(IRI-Fs)	17.38 (5.12)	1–28	0.75
Empathy—(IRI-PT)	19.20 (4.60)	7–28	0.76
Empathy—(IRI-PD)	8.98 (4.72)	0–21	0.76
Empathy—(IRI-EC)	21.34 (3.94)	9–28	0.70
Psychological well-being (PWBS)	93.91 (11.31)	61–122	0.72
Subjective well-being (WHO-5)	16.45 (4.48)	4–25	0.83

Fs = Fantasy; PT = Perspective Taking; PD = Personal Distress; EC = Empathic Concern.

**Table 3:** Results of Hierarchical Linear Regression Analyses.

	Outcome Variable					
	Psychological Well-Being (PWBS)			Subjective Well-Being (WHO-5)		
Independent Variable	Adjust. *R*^2^	Δ*R*^2^	*β*	Adjust. *R*^2^	Δ*R*^2^	*β*
Step 1	0.33 ***			0.15 ***		
Self-efficacy (PPEV)			0.39 ***			0.32 ***
Optimism (LOT-R)			0.33 ***			0.15 *
Step 2	0.35 ***	0.02 *		0.19 ***	0.05 **	
Self-efficacy (PPEV)			0.33 ***			0.23 **
Optimism (LOT-R)			0.30 ***			0.11
Empathy—(IRI-PT)			0.16 *			0.25 **
Step 3	0.36 ***	0.02 *		0.20 ***	0.02	
Self-efficacy (PPEV)			0.27 ***			0.18 *
Optimism (LOT-R)			0.29 ***			0.10
Empathy—(IRI-PT)			0.14 *			0.24 **
Empathy—(IRI-PD)			−0.16 *			−0.14

PT = Perspective Taking; PD = Personal Distress; *β* = standardized beta value. * *p* < 0.05. ** *p* < 0.01. *** *p* ≤ 0.001.

## Appendix A

**Table A1:** Pearson’s correlations between the study psychological variables.

	1	2	3	4	5	6	7
1. Self-efficacy (PPEV)	-						
2. Optimism (LOT-R)	0.31 ***	-					
3. Empathy (IRI-Fs)	0.09	0.18 *	-				
4. Empathy (IRI-PT)	0.43 ***	0.28 ***	0.27 ***	-			
5. Empathy (IRI-EC)	0.24 **	0.17 *	0.36 ***	0.31 ***	-		
6. Empathy (IRI-PD)	−0.45 ***	−0.19 *	0.09	−0.27 ***	0.05	-	
7. Psychological well-being (PWBS)	0.49 ***	0.45 ***	0.02	0.38 ***	0.15	−0.38 ***	-
8. Subjective well-being (WHO-5)	0.37 ***	0.25 ***	0.06	0.38 ***	0.10	−0.30 ***	0.41 ***

Fs = Fantasy; PT = Perspective Taking; EC = Emphatic Concern; PD = Personal Distress. * *p* < 0.05. ** *p* < 0.01. *** *p* ≤ 0.001.
